# Spectrophotometric Color Measurement to Assess Temperature of Exposure in Cortical and Medullar Heated Human Bones: A Preliminary Study

**DOI:** 10.3390/diagnostics10110979

**Published:** 2020-11-20

**Authors:** Leticia Rubio, Ramona Díaz-Vico, Inés Smith-Fernández, Aníbal Smith-Fernández, Juan Suárez, Stella Martin-de-las-Heras, Ignacio Santos

**Affiliations:** 1 Department of Forensic and Legal Medicine, Instituto de Investigación Biomédica de Málaga-IBIMA (CE-18), University of Malaga, 29071 Malaga, Spain; ramonadiaz57@hotmail.com (R.D.-V.); isantos@uma.es (I.S.); 2 Department of Human Anatomy, University of Malaga, 29071 Malaga, Spain; inessf@uma.es (I.S.-F.); janibal@uma.es (A.S.-F.); 3 Unidad de Gestión Clínica de Salud Mental, Instituto de Investigación Biomédica de Málaga-IBIMA, Hospital Regional Universitario de Málaga, 29010 Malaga, Spain; juan.suarez@ibima.eu

**Keywords:** color measurement, spectrophotometer, high temperature, bone

## Abstract

Heated-bone color changes may provide information about temperature of exposure, with interest for anthropologists and forensic experts. The aim of this study was to assess heat-induced color changes by spectrophotometry in cortical and medullar human bones heated at different temperatures and times. CIELAB (International Commission on Illumination-LAB) color parameters (L*, a*, and b*) and whiteness (WI) and yellowness (YI) indexes were obtained by spectrophotometry in the cortical and medullar zones of 36 bone sections exposed at 200, 400, 600, and 800 °C for 30 and 60 min. The accuracy of color-based temperature estimations was evaluated by Receiver Operating Characteristics (ROC) analysis. Chromaticity a* showed the best significant discrimination power with the area under the ROC curve (AUC) values ranged from 0.9 to 1.0 in cortical zones and 0.7 to 1.0 in medullar zones for all temperatures of exposures and both time of exposures. Chromaticity b*, and WI and YI indexes showed an AUC of 1.0 at 400, 600, and 800 °C for 30 and 60 min in the cortical and medullar zones. The spectrophotometric color parameters provided a highly accurate estimation of the temperature of exposure to discriminate between temperatures and exposure times in the cortical and medullar zones. Spectrophotometric bone color measurement in cortical and medullar zones can be an objective and reproducible method to estimate the temperature of exposition, and it can be considered useful for forensic and anthropological purposes.

## 1. Introduction

Thermal bone alterations are routinely examined in anthropology and forensic laboratories. Heat exposure induced water and organic material evaporations as well as contraction of bone tissues and changed the structure, shape, and color of them [1]. Changes in the color of burnt bones can provide information on their recovery, trauma history, structural changes, DNA degradation, and the temperature to which they were exposed [2,3]. The study of these changes is necessary since burned remains can be found in a great range of contexts such as forensic, anthropological, paleontological, or archeological contexts [4,5,6,7,8,9,10,11]. In forensic casework, the temperature that human remains have been subjected to, can reveal information about the origin and nature of fires. Forensics can deal with heated bones in accidentally or deliberated accidents like mass disasters, car accidents, or homicide. Burned or heat-altered bone remains are a major constituent of the archaeological record. Their presence may provide information about past human behavior and culture. The degree of burning is a crucial parameter for interpreting possible fire-related human activities like human cremations [12,13]. Finally, paleontologists may have to study possible uses of fire in ancient cultures [14,15,16]. Therefore, it would be necessary to develop objective and highly accurate methods to estimate the temperature of exposition.

Heated-bone color changes have been often visually assessed by comparison with shade guides like Munssell system and described as ivory, yellow-white to brown-black (carbonized bone) and bluish grey-white (calcined bone) [1,4,17,18]. However, besides being a subjective method, post-incineration colors did not always correspond to the shades offered. Krap et al. [19] demonstrated that caution should be taken in visual temperature estimation of thermally induced color changes in bone and recommended further research to develop objective methods in order to achieve standard of scientific community. Other recent studies have proposed a range of methods to quantify color changes in heated bone samples, such as colorimetric, spectrophotometric, or spectrographic techniques [2,20,21,22].

In a previous study, we have demonstrated how spectrophotometer analysis could be used to estimate the temperature of exposure with high accuracy in heated human teeth [23,24]. In this research, we propose to estimate the temperature based on color changes in medullar and cortical fresh human bone zones accurately measured with a digital spectrophotometer. As far as we know, this is the first time that color is assessed by spectrophotometry in fresh human heated cortical and medullar bone zones. Thus, the aims of this study were (1) to assess heat-induced color changes in human bones heated at different temperatures and times, using spectrophotometry as a quantitative method of color measurement, and (2) to determine the accuracy of temperature exposure estimations based on color measurements in medullar and cortical bone zones. This method could be reliable to be applied in anthropological and forensic cases in human bone samples where fire is involved.

## 2. Materials and Methods

### 2.1. Human Bone Sampling

The femurs, tibias, radii, and ulnae of 1 fresh human cadaver were obtained from the Department of Human Anatomy of the School of Medicine, University of Malaga (Spain). The donor consisted of a male aged 67 with no diagnosis of bone cancer or other bone diseases.

The sample was obtained by means of donation to science and education with approval of the Ethics Committee for Research involving Human Subjects at the University of Malaga (Spain) (approval number: 78-2015-H; approval date: 9 December 2015). The study was conducted in accordance with the Declaration of Helsinki by the World Medical Association (64th WWA General Assembly, Fortaleza, Brazil, October 2013) and the Recommendation No. R (97) 5 of the Committee of Ministers to Member States of the Protection of Medical Data (1997Fi).

### 2.2. Sample Preparation

The diaphysis of the femurs, tibias, radii, and ulnae were defleshed by using a scalpel and sawn into approximately 5-cm-thick transverse sections using an electric bone saw immersed in a layer of water to reduce heating due to friction. Thirty-six sections were obtained, carefully cleaned of any soft remains, and washed with distilled water. Then, the 36 sections were equally distributed to get a representation of each type of bone (femur, tibia, radius, and ulna) into 9 experimental groups. The samples were preserved in a refrigerator at 4 °C until analysis.

### 2.3. Incineration

Samples were heated in a muffle furnace (Nabertherm LT 40/12, Nabertherm GmbH, Lilienthal, Germany) at temperatures of 200, 400, 600, and 800 ℃ for 30 and 60 min. One group of four samples were stored at room temperature (21 ℃) and not heated (control group).

The chamber furnace was preheated starting from room temperature (21 ℃) to the designated temperature. A heating rate of 10 °C/min was used. The corresponding group of bones were placed in 99.9% alumina crucibles. Then, bone sections were heated for 30 or 60 min for each range of temperature.

### 2.4. Color Analysis

**Figure 1:**
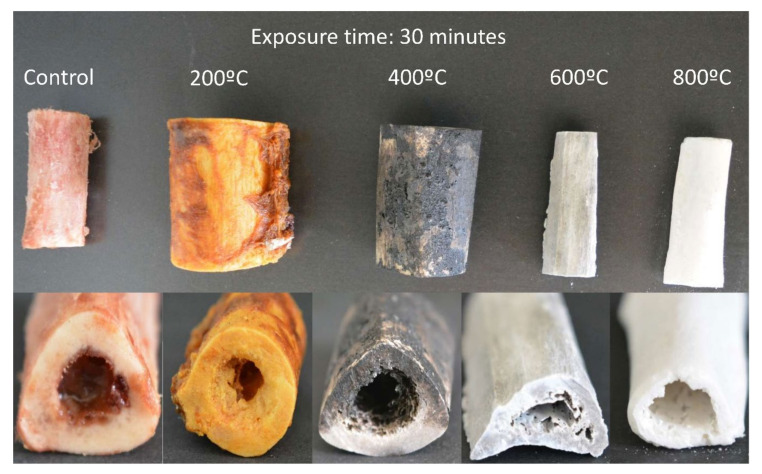
Color changes in bones after exposure at different temperatures (℃) for 30 min. From left to right: tibia, femur, tibia, ulna, and radius.

**Figure 2:**
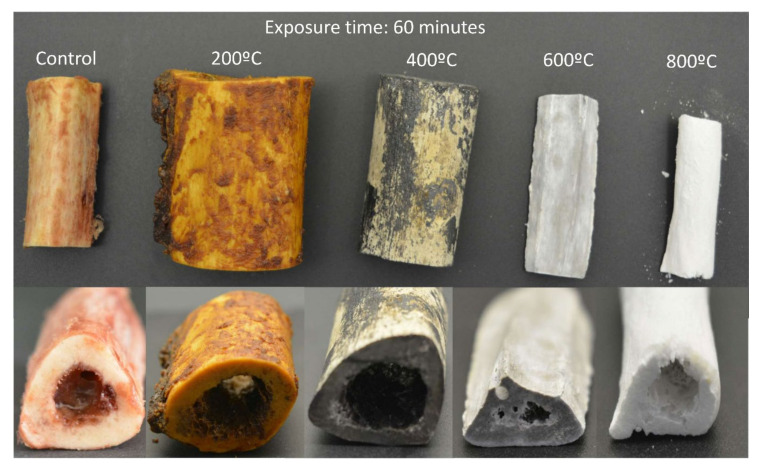
Color changes in bones after exposure at different temperatures (℃) for 60 min. From left to right: tibia, femur, tibia, ulna, and radius.

Immediately after exposure to experimental temperatures, each individual bone section was examined by an expert observer under natural light using a 2.5× magnification loupe (Orastic, Middleton, WI, USA) to analyze color visually. Visual inspection was supplemented with photographs taken with a digital reflex camera (Canon 500D^®^, Canon Inc., Tokyo, Japan), equipped with a 100-mm macro lens and a ring flash (Canon MR-14 Ex^®^, Canon Inc., Tokyo, Japan). The images were viewed with the Adobe Camera Raw 7.3^®^ program (Adobe Systems incorporated, San Jose, CA, USA) with the zoom mode up to 100%. Figure 1 and Figure 2 depict the representative bones after exposure at temperatures of 200, 400, 600, and 800 ℃ for 30 and 60 min.

Then, the color of the cortical and medullar zones of each bone section was determined by spectrophotometry using a portable contact Spectro-color^®^ device (Dr Lange, Keison Products Co., Chelmsford, England) with an 8-mm measuring tip in analysis mode. Device parameters were D65 for illuminant conditions and 8° for the standard observer. The spectrophotometer was calibrated before each measurement session. The mean of three measurements in three different sites was considered for each zone (cortical and medulla).

CIELAB color parameters (L*, a*, and b*) and the luminance (Y; units: cd/m^2^; candles/square meter) were measured. The L* value is a measure of the lightness of an object on a scale from 0 (black) to 100 (white). Chromaticity a* is a measure of redness (positive a*) or greenness (negative a*), and chromaticity b* is a measure of yellowness (positive b*) or blueness (negative b*). The a* and b* coordinates approach zero for neutral colors (white and grays) and increase in magnitude for more saturated or intense colors [25].

The whiteness and yellowness of bones were evaluated by calculating whiteness (WI) and yellowness (YI) indexes as proposed by the American Society of Testing Materials (ASTM) [26], using the following formulas:

(1) WI=4Z%−3Yrel

(2) $$YI=100\;\left(1-0.847\;Z/Yrel\right),$$

where *Z*% is calculated as $Z\%=\left(Z/Zn\right)100$, *Z* is the tristimulus *Z* of the sample, and *Zn* the tristimulus Z of the white standard, while *Yrel* is the luminance factor calculated as $Yrel=100\;\left(Y/Yn\right)$, where Y is the luminance of the sample and *Yn* is the luminance of the white standard.

### 2.5. Statistical Analysis

GraphPad Prism 7.05 (GraphPad Software, San Diego, CA, USA) was used for the statistical analysis. All data are represented as the mean ± standard error of the mean (SEM). Kolmogorov–Smirnov normality test indicated that all data followed a Gaussian distribution (*p* > 0.1); therefore, parametric statistics were applied. Groups were compared using one-way ANOVA and Bonferroni post hoc test for multiple comparisons (*p* < 0.05 was considered significant). To evaluate the effects (temperature, time, and bone area) and the interaction between them, a two-way ANOVA was performed. The accuracy of color-based temperature estimations was evaluated by receiver operating characteristics (ROC) analysis, which combines sensitivity and specificity in a single accuracy measure. Areas under the ROC curves (AUCs) between 0.5 and 1 indicate a positive relationship between the bone color parameter and temperature; 95% confidence intervals (CIs) were calculated.

## 3. Results

**Figure 3:**
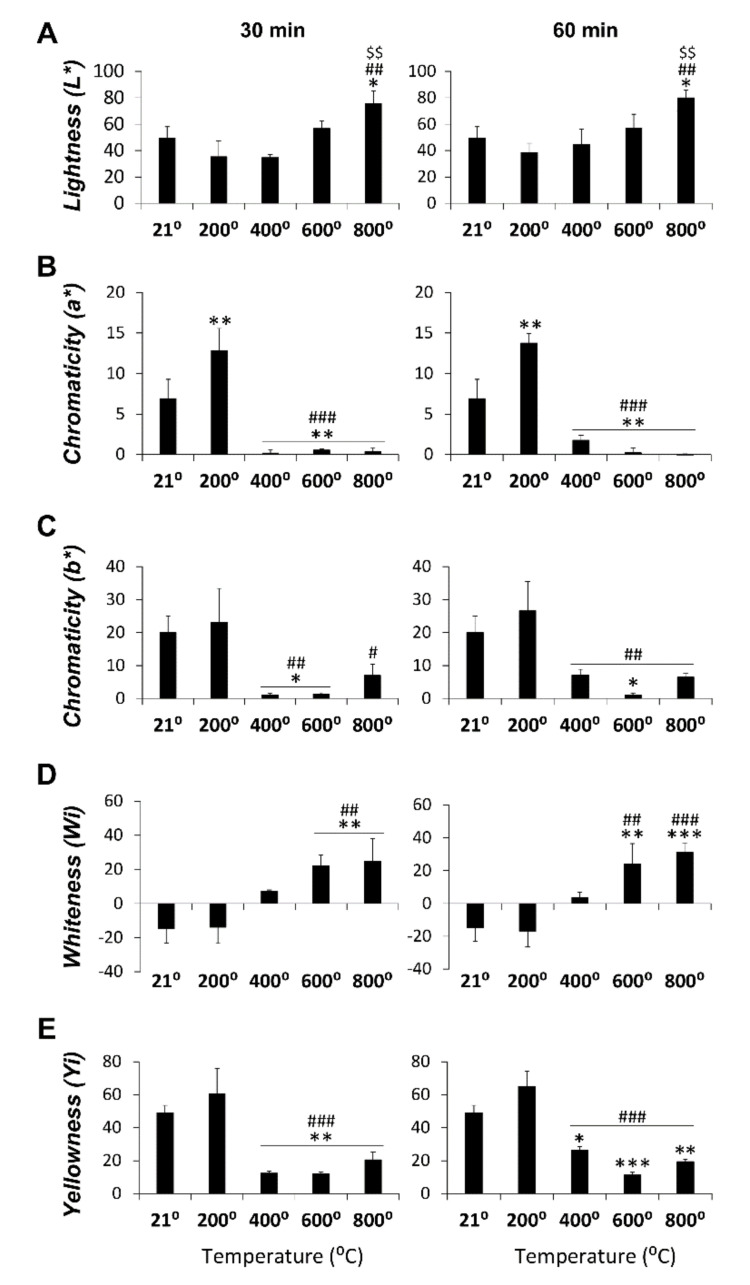
Spectrophotometric color measurements in cortical bone zone (L*, a*, b*, whiteness index (WI), and yellowness index (YI)) in the control and those heated at 200, 400, 600, and 800 ℃ for 30 and 60 min. (**A**): Lightness parameter for 30 and 60 min; (**B**): Chromaticity a* parameter for 30 and 60 min; (**C**): Chromaticity b* parameter for 30 and 60 min; (**D**): Whiteness parameter for 30 and 60 min and (**E**): Yellowness parameter for 30 and 60 min. Histograms represent means ± SEM (*n* = 4). ANOVA: *^/^**^/^*** *p* < 0.05/0.01/0.001 vs. control group, ^#/##/###^ *p* < 0.05/0.01/0.001 vs. 200 ℃, ^$$^ *p* < 0.01 vs. 400 ℃.

Figure 3 depicts statistical significance between temperature groups for all spectrophotometric parameters in the cortical zone at 30 min (main factor: F_4,14_ > 13.63, *p* < 0.0001) and 60 min (main factor: F_4,15_ > 13.01, *p* < 0.0001) of heat exposure. The highest L* value was obtained at 800 ℃ and was statistically significant versus the control group (21 ℃), 200, and 400 ℃ at 30 and 60 min of exposure (Figure 3A). The lower chromaticity a* values were found at 400, 600, and 800 ℃ and were statistically significant versus the control and 200 ℃ groups in both exposure times. In addition, the chromaticity a* values were the highest at 200 ℃ and statistically significant compared to the control group in both exposure times (Figure 3B). Chromaticity b* showed significant decreases at 400, 600, and 800 ℃ compared to the 200 ℃ group at 30 min and 60 min of exposure. In addition, the 400 and 600 ℃ groups at 30 min and the 600 ℃ group at 60 min also showed significant decreases compared to the control group (Figure 3C). WI showed significant increases at 600 and 800 ℃ compared to the remaining groups at 30 and 60 min of exposure (Figure 3D). The lower YI values were obtained at 400, 600, and 800 ℃ and were statistically significant to the control and 200 ℃ groups at 30 and 60 min (Figure 3E).

**Figure 4:**
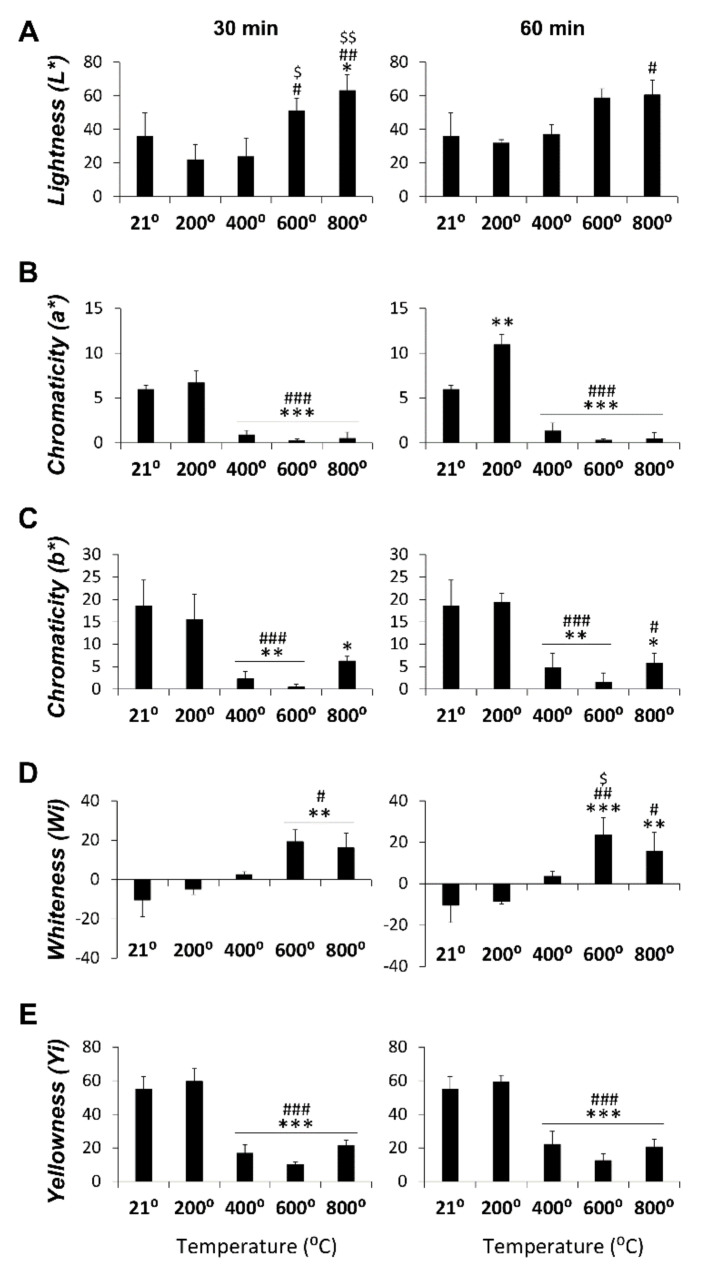
Spectrophotometric color measurements in the medullar bone zone (L*, a*, b*, WI, and YI) in the control and those heated at 200, 400, 600, and 800 ℃ for 30 and 60 min. (**A**): Lightness parameter for 30 and 60 min; (**B**): Chromaticity a* parameter for 30 and 60 min; (**C**): Chromaticity b* parameter for 30 and 60 min; (**D**): Whiteness parameter for 30 and 60 min and (**E**): Yellowness parameter for 30 and 60 min. histograms represent means ± SEM (*n* = 4). ANOVA: *^/^**^/^*** *p* < 0.05/0.01/0.001 vs. control group, ^#/##/###^ *p* < 0.05/0.01/0.001 vs. 200 ℃, ^$/$$^ *p* < 0.05/0.01 vs. 400 ℃.

Figure 4 depicts statistical significance between temperature groups for all spectrophotometric parameters in the medullar zone at 30 min (main factor: F_4,14_ > 11.13, *p* < 0.0003) and 60 min (main factor: F_4,15_ > 9.95, *p* < 0.0004) of exposure. L* values were statistically significant at 800 ℃ and 600 ℃ compared to the 200 ℃ group and at 400 ℃ and 800 ℃ compared to the control group at 30 min of exposition (Figure 4A). However, at 60 min, L* values significantly increased at 800 ℃ compared to the 200 ℃ group. The lowest significant values of chromaticity a* were observed at 400, 600, and 800 ℃ compared to the control and 200 ℃ groups in both exposure times. The highest chromaticity a* value was found at 200 ℃ at 60 min and was statistically significant compared to the control group (Figure 4B). The lowest significant values of chromaticity b* were detected at 400 and 600 ℃ versus the control and 200 ℃ groups in both exposure times. At 800 ℃, statistical significance with the control group at 30 min and with the 200 ℃ and control groups at 60 min were seen (Figure 4C). WI values at 30 min and 60 min of exposition showed statistical significance at 600 and 800 ℃ compared to the control and 200 ℃ groups and at 600 ℃ compared to the 400 ℃ group at 60 min (Figure 4D). The lowest YI values were found at 400, 600, and 800 ℃ and were statistically significant compared to the control and 200 ℃ groups in both exposure times (Figure 4E).

There is a main effect of temperature on all spectrophotometric parameters regardless of exposure time (30 and 60 min) in the cortical and medullar zones (F_4,30_ > 5.4, *p* < 0.002). Interaction between temperature and exposure time was only detected in the chromaticity a* of the medullar zone (F_4,30_ = 3.12, *p* = 0.029). Thus, the effect of exposure time on the spectrophotometric parameters was not observed in the bone zones. There is a main effect of temperature on the spectrophotometric parameters regardless of the bone zones (F_4,30_ > 5.57, *p* < 0.002). No interaction between temperature and bone zone (cortical and medulla) was observed in the spectrophotometric parameters in both exposure times (30 and 60 min). The single effect analysis only showed significant differences between cortical and medullar zones in chromaticity a* values at 200 ℃ for 30 min (** *p* < 0.01; Supplementary Figure S1C).

Table 1 shows the discriminative value of spectrophotometric parameters for each temperature studied in the cortical zone exposed for 30 and 60 min. At 30 min of exposure time, chromaticity a* values showed the best significant discrimination power with an AUC of 0.9 (95% CI; 0.7–1.0) at 200 ℃ and 1 (95% CI; 1.0–1.0) for the rest of temperatures and sensitivity and specificity values of 100/75% at 200 °C and 100/100% at the remaining temperatures. Chromaticity a* was followed by chromaticity b*, WI, and YI at 400, 600, and 800 ℃. At 60 min of exposure time, chromaticity a* showed the best significant discrimination power with an AUC of 1.0 (95% CI; 1.0–1.0) and sensitivity and specificity values of 100/75% at 200 ℃ and 100/100% at the remaining temperatures. Chromaticity a* was followed by YI at all temperatures and by chromaticity b* and WI at 400, 600, and 800 ℃ for 60 min of exposure time.

Table 2 shows the discriminative value of spectrophotometric parameters for each temperature studied in the medullar zone exposed for 30 and 60 min. For 30 min of exposure time, chromaticity a*, chromaticity b*, WI, and YI showed the best significant discrimination power with an AUC of 1.0 (95% CI; 1.0–1.0) and sensitivity and specificity values of 100/100% at 400, 600, and 800 ℃. For 60 min of exposure, chromaticity a* values showed an AUC of 1.0 (95% CI; 1.0–1.0) and a sensitivity and specificity value of 100/100% for all temperatures; followed by chromaticity b*, WI, and YI at 400, 600, and 800 ℃.

## 4. Discussion

In this study, highly accurate temperature of exposure predictions were obtained based on spectrophotometric measurement of colorimetric variables and other CIE (International Commission on Illumination)-recommended indexes in medulla and cortical zones of fresh bone fragments. To our best knowledge, this is the first time that the color of cortical and medullar areas have been spectrophotometric measured in heated bones. Spectrophotometric techniques avoid the subjectivity of color shade comparisons and utilize a white standard as reference with the same measurement conditions, and reproducibility of results has been successfully tested [23,24]. The main advantage of our study is that color measurement is performed directly on the bones, with no intermediate steps between sample and color measurements [22]. In addition, a major practical advantage of the contact spectrophotometric system used in this study is its portability, facilitating its routine use in practical forensic and anthropological cases.

High temperatures alter the chemical properties and structural integrity of bones through four stages: dehydration, decomposition, inversion, and fusion. Due to these changes, heat can produce fractures and bone will experiment measurable changes in dimensions, weight, porosity, crystallinity, and color [6,22]. Color bone changes due to heat are related to changes in the chemical composition of the organic components. When bone samples are exposed to heat in a muffle oven, the exposure to heat is uniform, which differs from the profound inhomogeneity of temperature in some real forensic and anthropological cases. Nevertheless, similar color changes can be expected in both experimental and real cases [20,22].

The strength of this study is that it has been carried out with fresh human bone samples. The use of nonhuman heated bones [2,13,21], including mammalian bone, could alter the color measurement. Mammalian bone is composed of the same chemical components, but there are differences in the proportion between organic and inorganic components. This could alter the heat-induced color changes since they are produced, among other factors, by the loss of organic matter [6]. The maximum temperature of fire and time of exposure of corpses vary quite significantly in the different forensic scenarios, such as explosions, where extremely high and fast temperatures or large time of exposure to fire with the intention to hide a corpse are reached. Furthermore, a body in a fire could be subjected to many different temperatures, depending on the origin and contributing factors. The time of heat exposure used in the current study was selected to embrace multiple circumstances in anthropological and forensic contexts [13,27,28], although some other studies have exposed bones to similar temperatures but for a shorter period of time [4,29,30,31].

Bone color changes after heat exposure can be influenced by a multitude of factors, including amount of oxygen and soft tissue present during heating, variation in soil composition or contaminants, specific skeletal elements exposed, and other related factors [9,21]. However, a recent study showed that sex, age, and skeletal elements had no significant contribution to the heat-induced color changes [22].

A diagnostic test is considered “highly accurate” with an AUC value of >0.9, “useful for some purposes” with a value of 0.7–0.9, and “poor” with one of 0.5–0.7 [32]. According to our results, chromaticity a* can be considered highly accurate to determine heat of exposure in the cortical zone at all temperatures for 60 min with a lower 95% CI limit of AUC > 0.9 (Table 1). Applying the same strict statistical interpretation, chromaticity a*, b*, WI, and YI parameters can be considered highly accurate to determine the heat of exposure in the cortical zone at 400, 600, and 800 ℃ for 30 and 60 min with a lower 95% CI limit of AUC > 0.9. Chromaticity a* is useful to determine 200 ℃ of heat exposure at 30 min with a lower 95% CI interval of AUC of 0.7 (Table 1 and Table 2). In the medullar zone (Table 2), chromaticity a*, chromaticity b*, WI, and YI can be considered highly accurate to determine the heat of exposure at 400, 600, and 800 ℃ for 30 and 60 min and chromaticity a* at 200 ℃ for 60 min, all of them with a lower 95% CI limit of AUC > 0.9. Hence, this method can be considered highly accurate to estimate the temperature of exposure of heated bones at 30 and 60 min in forensic and anthropological cases. For instance, when the chromaticity a* value is <2.865 in the cortical, the heat of exposure can be estimated at 400 ℃ at 30 min (AUC = 1.0) with a sensitivity and specificity of 100%.

The comparison of our results with previous findings is limited by the scant research about spectrophotometric color measurement in human bones. Most of the authors collected colorimetric data in an L* a* b* system [2,21,22,30,31], but only Wärmlander et al. [21] collected them by a spectrophotometer.

According to our results, spectrophotometric parameters discriminated with accuracy among temperatures and exposure times in the cortical and medullar zones. Chromaticity a*, chromaticity b*, and WI and YI indexes provided a highly accurate estimation of the temperature, with chromaticity a* being the most useful spectrophotometric color variable for its applicability at all temperature ranges studied, including at 200 ℃, and both times of exposure.

An important study limitation is that the sample size used was small, although from a single individual, avoiding the effect of the age and gender on the bone color. Another study limitation is that the effect of incineration was investigated in a controlled laboratory environment, and no account was taken of influential factor such as the type of accelerant involved or the method used to extinguish the fire. In addition, there was no exploration of the protective effect of soft tissues against high temperatures. Further studies are needed to analyze the effect of other factors on bone color such as gender and aging and the effect of fire on bones under different circumstances adding more times for heat exposition and a more gradual heating.

## 5. Conclusions

The spectrophotometric color parameters chromaticity a*, chromaticity b*, and WI and YI indexes provided a highly accurate estimate of the temperature of exposure of cortical and medullar human bones. Chromaticity a* was the most useful spectrophotometric variable due to its power of discrimination at lowest temperature (200 ℃). The discrimination model showed that all spectrophotometric parameters analyzed were useful to discriminate between temperatures and exposure times in the cortical and medullar zones, although limitations of the study should be taken into consideration. Spectrophotometric bone color measurement in cortical and medullar zones appears to offer an objective, reproducible, and useful approach to temperature of exposition estimation, and it can be considered useful for forensic and anthropological purposes.

## Figures and Tables

**Table 1:** Receiver Operating Characteristics (ROC) analysis predicting the temperature of burned cortical bone for 30 and 60 min with L, chromaticity a*, and chromaticity b* values and with whiteness (WI) and yellowness (YI) indexes.

Exposure Time: 30 min		Temperature (℃)			
		200	400	600	800
Lightness (L)	AUC ^a^ (95% CI)	0.8 (0.4–1.0)	0.8 (0.4–1.0)	0.7 (0.3–1.0)	1.0* (1.0–1.0)
	Youden ^b^	<44.24	<42.53	>47.79	>56.39
	Se ^c^/Sp ^d^	75/75	100/75	100/75	100/75
Chromaticity a*	AUC ^a^ (95% CI)	0.9* (0.7–1.0)	1.0* (1.0–1.0)	1.0* (1.0–1.0)	1.0* (1.0–1.0)
	Youden ^b^	>7.71	<2.865	<2.870	<2.895
	Se ^c^/Sp ^d^	100/75	100/100	100/100	100/100
Chromaticity b*	AUC ^a^ (95% CI)	0.7 (0.3–1.0)	1.0* (1.0–1.0)	1.0* (1.0–1.0)	1.0* (1.0–1.0)
	Youden ^b^	<24.23	<8.685	<8.845	<13.73
	Se ^c^/Sp ^d^	75/75	100/100	100/100	100/100
Whiteness (WI)	AUC ^a^ (95% CI)	0.5 (0.1–0.9)	1.0* (1.0–1.0)	1.0* (1.0–1.0)	1.0* (1.0–1.0)
	Youden ^b^	>−20.1	>−0.50	>3.550	>3.05
	Se ^c^/Sp ^d^	75/50	100/100	100/100	100/100
Yellowness (YI)	AUC ^a^ (95% CI)	0.7 (0.3–1.0)	1.0* (1.0–1.0)	1.0* (1.0–1.0)	1.0* (1.0–1.0)
	Youden ^b^	>52.43	<28.72	< 28.66	<35.21
	Se ^c^/Sp ^d^	75/75	100/100	100/100	100/100
**Exposure Time: 60 min**		**Temperature (℃)**			
		**200**	**400**	**600**	**800**
Lightness (L)	AUC ^a^ (95% CI)	0.8 (0.6–1.0)	0.5 (0.1–0.9)	0.6 (0.2–1.0)	1.0* (1.0–1.0)
	Youden ^b^	<45.36	<51.44	>47.51	>56.39
	Se ^c^/Sp ^d^	100/75	75/50	100/50	100/75
Chromaticity a*	AUC ^a^ (95% CI)	1.0* (1.0–1.0)	1.0* (1.0–1.0)	1.0* (1.0–1.0)	1.0* (1.0–1.0)
	Youden ^b^	>8.235	<3.715	<2.970	<2.605
	Se ^c^/Sp ^d^	100/75	100/100	100/100	100/100
Chromaticity b*	AUC ^a^ (95% CI)	0.6 (0.2–1.0)	1.0* (1.0–1.0)	1.0* (1.0–1.0)	1.0* (1.0–1.0)
	Youden ^b^	>23.09	<12.31	<8.835	<11.59
	Se ^c^/Sp ^d^	75/75	100/100	100/100	100/100
Whiteness (WI)	AUC ^a^ (95% CI)	0.6 (0.1–1.0)	1.0* (1.0–1.0)	1.0* (1.0–1.0)	1.0* (1.0–1.0)
	Youden ^b^	<−21.95	>−2.750	>4.600	<28.44
	Se ^c^/Sp ^d^	50/75	100/100	100/100	100/100
Yellowness (YI)	AUC ^a^ (95% CI)	0.9* (0.7–1.0)	1.0* (1.0–1.0)	1.0* (1.0–1.0)	1.0* (1.0–1.0)
	Youden ^b^	>51.39	<36.55	<28.44	<32.09
	Se ^c^/Sp ^d^	100/75	100/100	100/100	100/100

^a^ AUC: area under the ROC. ^b^ Youden: cutoff point that maximizes the sum of sensitivity and specificity. ^c^ Se: sensitivity. ^d^ Sp: specificity. * *p* < 0.05.

**Table 2:** Receiver Operating Characteristics (ROC) analysis predicting temperature of burned medullar bone for 30 and 60 min with L, chromaticity a*, and chromaticity b* values and with whiteness (WI) and yellowness (YI) indexes.

Exposure Time: 30 min		Temperature (℃)			
		200	400	600	800
Lightness (L)	AUC ^a^ (95% CI)	0.8 (0.4–1.0)	0.8 (0.4–1.0)	0.8 (0.6–1.0)	0.9* (0.7–1.0)
	Youden ^b^	<31.07	<32.72	>38.67	>42.85
	Se ^c^/Sp ^d^	100/75	100/75	100/75	100/75
Chromaticity a*	AUC ^a^ (95% CI)	0.7 (0.3–1.0)	1.0* (1.0–1.0)	1.0* (1.0–1.0)	1.0* (1.0–1.0)
	Youden ^b^	>6.30	<3.395	<2.945	<3.40
	Se ^c^/Sp ^d^	75/75	100/100	100/100	100/100
Chromaticity b*	AUC ^a^ (95% CI)	0.6 (0.1–1.0)	1.0* (1.0–1.0)	1.0* (1.0–1.0)	1.0* (1.0–1.0)
	Youden ^b^	<21.46	<8.120	<6.875	<10.09
	Se ^c^/Sp ^d^	100/50	100/100	100/100	100/100
Whiteness (WI)	AUC ^a^ (95% CI)	0.6 (0.1–1.0)	1.0* (1.0–1.0)	1.0* (1.0–1.0)	1.0* (1.0–1.0)
	Youden ^b^	>−9.25	>−1.250	>3.350	>0.50
	Se ^c^/Sp ^d^	100/50	100/100	100/100	100/100
Yellowness (YI)	AUC ^a^ (95% CI)	0.6 (0.1–1.0)	1.0* (1.0–1.0)	1.0* (1.0–1.0)	1.0* (1.0–1.0)
	Youden ^b^	>48.87	<32.20	<28.14	<35.00
	Se ^c^/Sp ^d^	100/25	100/100	100/100	100/100
**Exposure Time: 60 min**		**Temperature (℃)**			
		**200**	**400**	**600**	**800**
Lightness (L)	AUC ^a^ (95% CI)	0.6 (0.2–1.0)	0.5 (0.1–0.9)	0.9* (0.7–1.0)	0.9* (0.7–1.0)
	Youden ^b^	<45.36	<51.44	>47.51	>56.39
	Se ^c^/Sp ^d^	100/50	75/50	100/50	100/75
Chromaticity a*	AUC ^a^ (95% CI)	1.0* (1.0–1.0)	1.0* (1.0–1.0)	1.0* (1.0–1.0)	1.0* (1.0–1.0)
	Youden ^b^	>8.235	<3.715	<2.970	<2.605
	Se ^c^/Sp ^d^	100/100	100/100	100/100	100/100
Chromaticity b*	AUC ^a^ (95% CI)	0.6 (0.1–1.0)	1.0* (1.0–1.0)	1.0* (1.0–1.0)	1.0* (1.0–1.0)
	Youden ^b^	>23.09	<12.31	<8.835	<11.59
	Se ^c^/Sp ^d^	100/50	100/100	100/100	100/100
Whiteness (WI)	AUC ^a^ (95% CI)	0.5 (0.09–0.9)	1.0* (1.0–1.0)	1.0* (1.0–1.0)	1.0* (1.0–1.0)
	Youden ^b^	<−21.95	>−2.750	>4.600	<28.44
	Se ^c^/Sp ^d^	100/50	100/100	100/75	100/100
Yellowness (YI)	AUC ^a^ (95% CI)	0.6 (0.1–1.0)	1.0* (1.0–1.0)	1.0* (1.0–1.0)	1.0* (1.0–1.0)
	Youden ^b^	>51.39	<36.55	<28.44	<32.09
	Se ^c^/Sp ^d^	100/50	100/100	100/100	100/100

^a^ AUC: area under the ROC. ^b^ Youden: cutoff point that maximizes the sum of sensitivity and specificity. ^c^ Se: sensitivity. ^d^ Sp: specificity. * *p* < 0.05.

## Supplementary Materials

The following are available online at https://www.mdpi.com/2075-4418/10/11/979/s1, Figure S1. Spectrophotometric color measurements in cortical and medullar bone zones (L*, a*, b*, WI, and YI) in the control and those heated at 200, 400, 600, and 800 ℃ for 30 and 60 min.
