# Supplementary material for: Spectrophotometric Color Measurement to Assess Temperature of Exposure in Cortical and Medullar Heated Human Bones: A Preliminary Study
Source: Diagnostics (Basel). 2020 Nov 20;10(11):979. doi: 10.3390/diagnostics10110979 (PMC7699973; doi:10.3390/diagnostics10110979)
Supplement: Supplementary file 1 [file diagnostics-10-00979-s001.pdf]

Supplementary figure 1

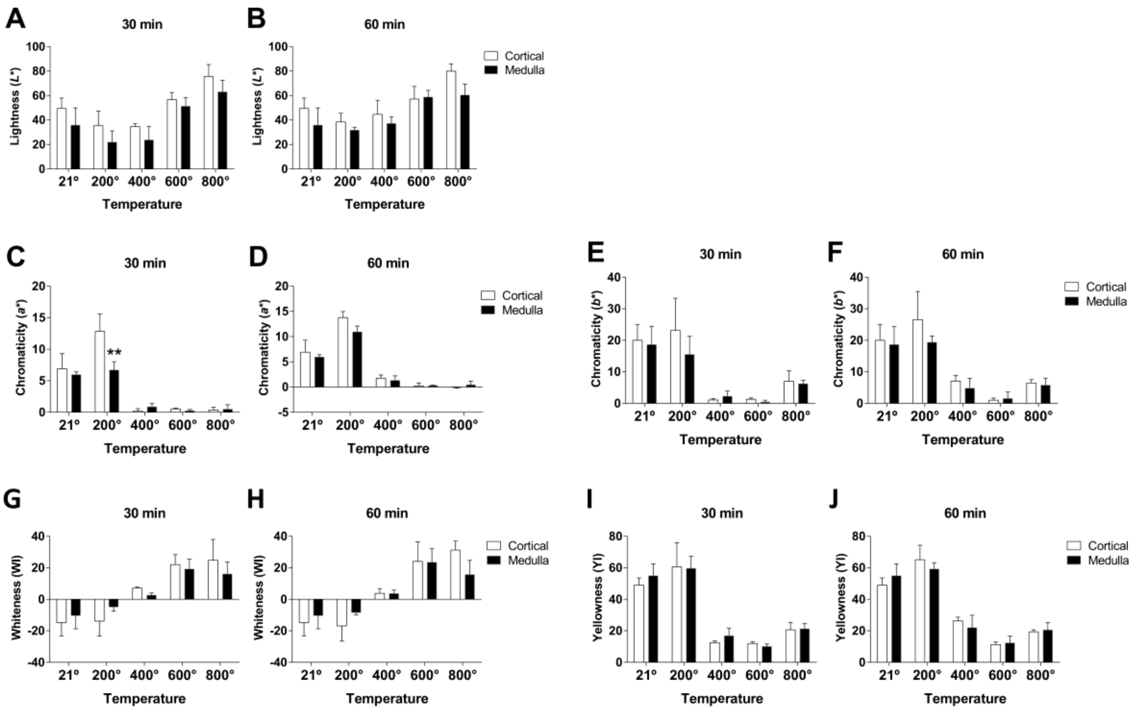

Figure S1. Spectrophotometric color measurements in cortical and medullar bone zones (L*, a*, b*, WI and YI) in control and those heated at 200, 400, 600 and 800 °C for 30 and 60 min. Histograms represent means ± SEM (n = 4). ANOVA: **p<0.01 *vs*. control group.
